# ‘Highlighting a gap in radiographers' pain knowledge: A letter to the editor on ‘Radiographers’ decision‐making processes in the movement of pain‐afflicted adult trauma patients in planar diagnostic imaging’’

**DOI:** 10.1002/jmrs.692

**Published:** 2023-06-01

**Authors:** Sophie Shephard, Kate Dahlenburg

**Affiliations:** ^1^ School of Allied Health, Exercise and Sports Sciences Charles Sturt University New South Wales Wagga Wagga Australia; ^2^ Department of Medical Imaging Logan Hospital Queensland Meadowbrook Australia

**Keywords:** imaging department, pain, radiography

## Abstract

This letter to the editor highlights a crucial knowledge gap within the radiography profession—a modern understanding of pain. The outdated belief that pain is directly correlated to tissue injury or pathology is misleading and potentially harmful, as it could lead to the stigmatisation and invalidation of patients with pain. Radiographers are urged to listen to their patients in order to truly understand their pain, and to embrace the opportunity to develop their knowledge and enhance the experience of patients in the imaging department.

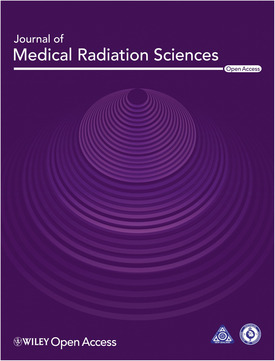

We commend Talevski et al. for their insightful paper^1^ and for bringing attention to the important issue of pain, which is a primary complaint for up to 78% of people presenting to emergency departments.^2^ Their research highlights that radiographers use a variety of approaches to improve patient comfort, however, a quote from one radiographer has captured what may represent a critical knowledge gap within the radiography profession: a modern understanding of pain.

I would say that often the actual pathology or the level of the injury often dictates how much pain the patient's going to be in^1^ p.6.

The understanding reflected in this quote is consistent with historical models of pain, where pain was thought to be the direct result of tissue injury or pathology, something ‘detected’ in the body tissues.^3^ These beliefs remain pervasive amidst health professionals, despite extensive research refuting this model.^4^, ^5^ The neurobiology of pain is complex, and beyond the scope of this letter, though it is vital to emphasise that tissue injury and pain are in fact often poorly correlated.^3^, ^6^ Rather than being simply the direct result of tissue pathology, pain can be more accurately conceptualised as a protective output of the nervous system, the product of the complex interplay of a vast array of biological, psychological and social factors.^7^ Pain is able to occur in the absence of tissue pathology,^3^ and conversely, many ‘pathological’ musculoskeletal imaging findings have been found to be present in a large percentage of asymptomatic individuals,^7^, ^8^, ^9^ highlighting that the link between tissue pathology and pain can be tenuous. While it is clear that radiographers do also use patient reports of pain to inform positioning,^1^ using imaging findings to determine a person's level of pain may be problematic, as patients who do not have an obvious cause for their level of symptoms on imaging or other objective clinical tests may be disbelieved,^4^ or even labelled a ‘hypochondriac’ or ‘drug‐seeker’.^10^, ^11^, ^12^ There is a risk that if radiographers do not believe someone is truly in pain based on their imaging findings, they may make harmful judgements, or be less empathetic or sensitive in the positioning of patients as they otherwise would be. Understanding pain is therefore crucial in order to avoid unintentionally stigmatising and invalidating patients with pain, and to ensure compassionate, person‐centred care in the imaging department.

Radiographers have a clear desire to place patient comfort at the forefront of their approach to imaging examinations, though this endeavour could be impeded by an outdated understanding of pain. While radiographers should continue to utilise their skills in image interpretation to assess if patient positioning is safe, imaging findings alone cannot determine how much pain someone is experiencing: the only way this can be known is by listening to the patient. We thank the authors for their research and believe they have captured a valuable opportunity to develop radiographers' knowledge and improve the experiences of patients presenting with pain in the imaging department.

## Funding Statement

This research was not supported by funding.

## Conflict of Interest Statement

The authors disclose no conflicts of interest.

## Data Availability

Data sharing not applicable to this article as no datasets were generated or analysed during the current study.
